# Shengxian decoction alleviates cyclophosphamide-induced immunosuppression via improving B cell-mediated immune responses

**DOI:** 10.3389/fphar.2025.1565451

**Published:** 2025-04-23

**Authors:** Chengyu Yang, Ya Xu, Fenfang Gong, Silu Li, Huang Zhan, Zhixuan Huang, Maolin Wang, Hui Li, Hengjun Huang

**Affiliations:** ^1^ Jiangxi Province Key Laboratory of Traditional Chinese Medicine Pharmacology, Institute of Traditional Chinese Medicine Health Industry, China Academy of Chinese Medical Sciences, Nanchang, China; ^2^ Jiangxi Health Industry Institute of Traditional Chinese Medicine, Nanchang, China; ^3^ Institute of Chinese Materia Medica, China Academy of Chinese Medical Sciences, Beijing, China

**Keywords:** Shengxian decoction, cyclophosphamide, immunosuppression, B cell, humoral immune responses

## Abstract

**Background:**

Chemotherapy is a prevalent and extensively utilized cancer treatment modality. However, it can result in immunosuppression. Shengxian Decoction (SXD) is a Traditional Chinese Medicine formula that has been demonstrated to improve immunosuppression caused by chemotherapy in clinical settings. Nevertheless, the therapeutic evaluation and mechanism of SXD in regulating immunosuppression remain unclear. The aim of this study was to ascertain the efficacy of SXD in immunocompromised mice and to elucidate the underlying immunological mechanisms.

**Methods:**

The immunosuppression mouse model was generated through the administration of cyclophosphamide for 3 days. After that, the mice were treated by SXD extracts at doses of 0.47 and 0.94 g/kg/day, respectively. The spleen, thymus and blood of mice were collected for evaluation of drug efficacy. The population of B and T cells was detected by flow cytometry. The genes regulated by SXD in spleen were identified by utilizing RNA sequencing. The Gene Ontology and Kyoto Encyclopedia of Genes and Genomes enrichment were used to analyze the signaling pathway modulated by SXD. The protein expression of B cell transcription factors was detected by the immunoblotting. The signaling pathways modulated by SXD were elucidated using transcriptomics analysis complemented by network analysis.

**Results:**

SXD treatment ameliorates decreased splenic and thymic organ indexes, as well as decreased lymphocyte number in the spleen in immunosuppressed mice. ELISA assay and flow cytometry shows that SXD promotes attenuate B-cell-mediated humoral immune responses. The RNA sequencing reveals that SXD primarily upregulates the B cell-mediated immune response and B cell receptor signaling pathway. SXD treatment upregulates the expression of B cell differentiation factors Pax5, Tcf3 and promotes splenic cell proliferation. Furthermore, SXD extract significantly inhibits hypoxia and senescence pathways in the spleen.

**Conclusion:**

SXD exerts a protective effect against CTX-induced immunosuppression by upregulating B cell immunity and inhibiting hypoxia and senescence pathways.

## 1 Introduction

Chemotherapy is a common and widely utilized cancer treatment. Cyclophosphamide (CTX) is a chemotherapeutic agent employed in the treatment of hematologic and lymphatic malignancies (Emadi et al., 2009). However, CTX has been demonstrated to induce immunosuppression and impair both cellular and humoral immune responses (Hurd and Giuliano, 1975; Huyan et al., 2011; Shirani et al., 2015). Furthermore, previous studies have demonstrated that CTX significantly reduces the number of B cells, impairs B-cell proliferation and antibody production (Sabbele et al., 1988; Zandvoort et al., 2001). B cells play a pivotal role in humoral immune responses, undergoing differentiation into plasma cells that produce immunoglobulins (Igs) (Wang et al., 2020). The development and proliferation of B cells is dependent on the expression of numerous transcription factors, including Pax5, Tcf3 (encoding E2A) and Myc (Kwon et al., 2008; Luo et al., 2018; Calderón et al., 2021). Pax5 and Tcf3 are indispensable for B cell differentiation, maintenance, and antibody diversity (Medvedovic et al., 2011; Boast et al., 2023). Myc is a master regulator of B cell proliferation and the formation of germinal center (GC) B cells (Finkin et al., 2019). It is anticipated that the number of B cells and antibody levels will be increased by regulating these transcription factors, thereby reversing the humoral immune damage induced by CTX.

In addition to the impact of transcription factors, the homeostasis of B lymphocytes is also regulated by hypoxia and senescence. The deletion of the pVHL gene has been demonstrated to result in overaccumulation of Hif-1α, which in turn leads to the absence of peripheral B-cell number and a reduction in antibody diversity (Burrows et al., 2020). Over-activation of Hif-1α has also been demonstrated to downregulate the B-cell receptor signaling pathway and to inhibit B cell maturation (Xu et al., 2019; Burrows et al., 2020). Overexpression of Hif-1α in GC B cells resulted in impaired GC B cell responses, reduced antigen-specific binding of antibodies (Cho et al., 2016). Aging is associated with a number of changes in the body, including increased differentiation of myeloid cells, a decrease in the proportion of lymphocytes, and elevated chronic inflammation (Li X. et al., 2023). It has been demonstrated that senescent immune cells play a pivotal role in the process of systemic ageing, particularly with regard to the acceleration of solid organ ageing (Yousefzadeh et al., 2021). Therefore, improving the aging state of immune cells helps to enhance immunity.

In the theoretical framework of traditional Chinese medicine (TCM), immunosuppression is primarily attributed to the stagnation of “qi” and the deficiency of “defensive qi” (Bi et al., 2024). The Shengxian decoction (SXD), a renowned TCM formula, was developed by the esteemed Chinese medicine master Zhang Xi-chun (1860–1933). The SXD formula is comprised of five botanical drugs: Astragali Radix (HuangQi), Bupleuri Radix (ChaiHu), Cimicifugae Rhizoma (ShengMa), Anemarrhenae Rhizoma (ZhiMu), and Platycodonis Radix (JieGeng). SXD has been demonstrated to have the beneficial effect of raising “qi” and is used to treat “atmospheric depression” syndrome in China. In accordance with the TCM theory, SXD has been employed in some clinical cases to alleviate immunosuppression resulting from chemotherapy (Jiang et al., 2022). Nevertheless, the efficacy and mechanism of SXD in improving immunosuppression are still unclear.

In this study, we systematically evaluated the effect and mechanism of SXD on regulating adaptive immune cell in CTX-induced immunocompromised mice. The administration of SXD can significantly improve the decrease of spleen index and the number of spleen immune cells induced by CTX. RNA sequencing revealed that SXD predominantly upregulated the B cell receptor (BCR) pathway and B-cell transcription factors Pax5 and Tcf3. Additionally, SXD can also promote the proliferation of spleen cells. Moreover, SXD treatment elevated the proportion of intestinal germinal center B cells and facilitated the production of class-switched antibodies, which are pivotal for humoral immunity. Furthermore, the targets of SXD significantly inhibited hypoxia and senescence signaling pathways. Therefore, SXD improves CTX-induced immunosuppression by promoting B-cell-mediated humoral immunity.

## 2 Materials and methods

### 2.1 Reagents

*Astragali radix* [Fabaceae; Astragalus membranaceus var. mongholicus (Bunge) Hsiao] (batch No. 2302003), *Anemarrhena Rhizoma* [Liliaceae; Anemarrhena asphodeloides Bge.] (batch No. 2310001), Bupleuri Radix [Apiaceae; Bupleurum Chinese DC.] (batch No. 2304001), Platycodonis Radix [Campanulaceae; Platycodon grandiflorum (Jacq.) A. DC.] (batch No. 2202001) and Cimicifugae Rhizoma [Ranunculaceae; Cimicifuga foetida L.] (batch No. 2308001) were purchased from Anguo Changda Chinese herbal medicine pieces Co., LTD. (Anguo, China). The botanical drugs were identified according to Pharmacopoeia of the People’s Republic of China 2020 Edition. Platycodin D (batch No. 16995, 98.8%), Astragaloside IV (batch No. 16275, 100.0%), Isoferulic acid (batch No. 14849, 99.5%), Saikosaponin A (batch No. 14977, 98.1%), Timsaponin B II (batch No. 15312, 99.5%) were purchased from Shanghai Standard Technology Co., Ltd. (Shanghai, China). Cyclophosphamide (CTX) was purchased from the Shanghai Yuanye Bio-Technology Co.,Ltd. (Shanghai, China). Lipopolysaccharide (LPS) was purchased from Sigma-Aldrich Co., Ltd. (Michigan, United States). The anti-CD3 (FITC), anti-CD4 (APC), anti-CD19 (PE-Cy7) and FAS (APC) antibodies were purchased from Thermo Fisher Scientific (Waltham, MA, United States). The anti-CD38 (PerCP-Cy5.5), anti-CD8 (PE) antibodies were provided from Biolegend (San Diego, CA). The anti- E2A Rabbit pAb (A22543), anti- PAX5 Rabbit pAb (A3068) antibodies were provided from ABClonal (Wuhan, China). The HRP-conjugated GAPDH Monoclonal antibody (HRP-60004) was purchased from Proteintech (Wuhan, China). Mouse IgA, IgG and IgM ELISA kit were purchased from Beyotime (Shanghai, China), Levamisole hydrochloride (LH) was produced from Renhe Church Pharmaceutical Co., Ltd. (Shandong, China). The cell counting kit (CCK-8) was purchased from Beyotime Biotechnology (Shanghai, China).

### 2.2 SXD preparation

SXD was prepared according to the previous report with modified (Li et al., 2022). Briefly, the five crude botanical drugs (Astragali Radix 18 g, Anemarrhena Rhizoma 3 g, Bupleuri Radix 4.5 g, Platycodonis Radix 4.5 g and Cimicifugae Rhizoma 3 g) soaked in 2000 mL distilled water and boiled with reflux for 30 min, then the decoction is filtered and collected. The residues were treated again under the same conditions. The sum of the decoctions was combined and concentrated using reduced pressure (−0.07∼−0.08 MPa) at 70°C. SXD extracts were freeze-dried for gaining lyophilized powders (26.1% extraction rate) for further studies.

### 2.3 SXD chemical metabolites analysis

Characterization of main chemical metabolites in SXD was performed on an ExionLCTM AC system (AB SCIEX), which coupled to a X500R QTOF system (AB SCIEX) via a TurboV ion source with electrospray ionisation (ESI) interface. Chromatographic separation was achieved on a Waters HSS T3 column at a flow rate of 300 μL/min. The mobile phase consisted of 0.1% formic acid water (A) and acetonitrile (B) and the elution gradient was set as follows: 5% B (0–1.5 min), 5%–20% B (1.5–5 min), 20%–35% B (5–12 min), 35%–40% B (12–16 min), 40%–70% B (16–19 min), 70%–90% B (19–20 min), 90% B (20–22 min), 90%–5% B (22–24 min), 5% B (24–26 min). The injection volume was 2.0 μL and the column temperature was 40°C. The mass spectra were acquired in negative ion mode. The optimized MS conditions were as follows: gas 1, 55 psi; gas 2, 55 psi; curtain gas, 35; temperature, 550°C; declustering potential, −60 V; collision energy, −35 V; CE spread, 10 V. The mass range was set as 100–1,500 Da of TOF MS and 50–1,500 Da of TOF MS/MS. A dynamic background subtraction based on information dependent acqui-sition (IDA) experiment was applied for MS/MS experiment and the exclude former candidate ions was set for 6 s after 2 occurrences. Data acquisition, processing, analysis was performed using SCIEX OS software (V3.0, AB SCIEX) and metabolites matching was achieved through a self-building database containing mass spectrometry information of reported metabolites from each botanical drug.

In addition, HPLC analysis was also used to analyze the metabolites of SXD with five standard metabolites. SXD extracts were dissolved in ethanol (8 mg/mL) and sonicated for 30 min in the ice-water bath. The supernatant was filtered through a 0.22 μm microporous membrane for HPLC analysis. The standard solutions were prepared as follow: proper amount of isoferulicacid, timosaponin BⅡ, platycodin D, astragaloside Ⅳ and saikosaponin A were precisely weighed and placed in a 2 mL volumetric flask. The mixed solutions containing isoferulicacid 0.49 mg, timosaponin BⅡ 0.49 mg, platycodin D 0.47 mg, astragaloside Ⅳ 0.44 mg and saikosaponin A 0.48 mg per 1 mL was prepared by methanol. The solutions were filtered through a 0.22-μm filter prior to HPLC analysis. HPLC analysis was performed on the Waters Alliance e2695 with an Agilent SB-C_18_ column (4.6 mm × 250 mm, 5 µm) and AllChrom 6100 evaporative light scattering detector. The flow rate was set at 1.0 mL/min. The drift tube temperature of evaporative light scattering detector was set at 112.8°C and the nitrogen flow rate of evaporative light scattering detector was set 3.1 L/min. The mobile phase consisted of acetonitrile (A) and water (B). The multi-step linear elution gradient program was as follows: 0–10 min, 5%–18% A; 10–16 min, 18%–23% A; 16–26 min, 23%–24% A; 26–36 min, 24%–31% A; 36–42 min, 31%–37% A; 42–55 min, 37%–44% A; 55–65 min, 44%–55% A; 65–70 min, 55%–5% A.

### 2.4 Animals and cyclophosphamide-induced immunosuppression mouse model

Male C57BL/6 mice of 6–8 weeks old (18–22 g) were purchased from SPF (Beijing) Biotechnology Co., Ltd. (Beijing, China. License number: SCXK 2019-0010). The mice were kept in the animal facility with a 12 h light/dark cycle and were provided food and water. All animal experiments were approved by the Committee on Animal Care and Use of the Institute of Traditional Chinese Medicine Health Industry, China Academy of Chinese Medical Sciences (Animal Ethics Committee approval number: 2024008).

The mice were randomly divided into five groups: the control (CL), the immunosuppression model group (ML), the LH administration group as positive control (PC), low and high SXD group (SXDL, SXDH). From day 1 to 3, mice in the CL group were treated once daily with 0.9% physiologic saline (i.p.), and the mice from other groups were treated once daily with cyclophosphamide (i.p.) at 80 mg/kg. From day 4 to day 10, mice in the CL and ML groups were treated once daily with 0.9% physiologic saline (i.g.); mice in the PC, SXDL and SXDH group were treated once daily with LH at 20 mg/kg (i.g.), SXD extracts at 0.47 g/kg and 0.94 g/kg (i.g., equivalent to the human equivalent dose) (Jiang et al., 2022), respectively. The body weights were recorded every other days.

### 2.5 Calculation of organ index

The thymus and spleen of each group were isolated and weighted. The thymus and spleen indexes were calculated by the following formula: $\text{Thymus or Spleen index }\left(\%\right)=\left(\text{spleen or thymus weight}/\text{body weight}\right)\times 100\%$ .

### 2.6 Hematoxylin and eosin (H&E) staining

The histopathological examination of spleens from each group was performed by H&E staining. In brief, after isolated from mice, spleens were fixed with 4% paraformaldehyde for 24 h, dehydrated with alcohol, embedded into paraffin and then cut into 3–5 mm sections. After dewaxing, these sections were incubated with hematoxylin for 5 min, then dehydrated by 100% alcohol for 1 min, and treated with eosin for 30 s. The sections were mounted and the images were acquired by light microscope (Leica DM750, Wetzlar, GER) and analyzed by CaseViewer software.

### 2.7 Immunohistochemistry (IHC) staining

The slices from spleen tissues with 5 μm thickness were dewaxed, hydrated, and performed antigen retrieval by microwaving in citrate buffer (10 mM, pH 6.0). After that, sections were blocked with 3% BSA (IA0910, Solarbio, Beijing, China), and rinsed with PBS, then incubated with anti-Ki67 antibodies (ab15580, Abcam, United States) at 4°C overnight. After washed by PBS, the slices were labelled with HRP-contained anti-rabbit antibody at room temperature for 1 h, and treated with DAB substrate solution (DA1016, Solarbio, Beijing, China). All images were observed with microscope and analyzed by using ImageJ software.

### 2.8 Lymphocyte proliferation

The spleens were isolated and washed by cold PBS, then passed through a 80 μm filter to obtain a splenic cell suspension. The erythrocytes were lysed and the remaining cells were centrifuged at 1,500 rpm for 5 min. After centrifugation, cells were resuspended in RPMI-1640 medium with 20% FBS, and then placed into 96-well plates in triplicate at a density of 5 × 10^6^ cells/mL. After that, the LPS (2 μg/mL) was added and followed by incubation for 48 h at 37°C in a humidified atmosphere containing 5% CO_2_. Then CCK-8 was added to each well, and after additional incubation for 2 h the absorbance at 450 nm was detected by utilizing a multimode microplate reader (VICTOR Nivo, Revvity, China).

### 2.9 Flow cytometry

Single-cell suspensions from spleens or mesenteric lymph nodes were obtained by removing red blood cells using RBC lysis buffer (Biolegend) and passing through a 200-mesh filter. After that, the indicated antibodies were used to stain T and B lymphocytes in PBS containing 5% (v/v) FBS at 4°C. The data were collected using CytoFLEX S flow cytometry analyzer (Beckman Coulter). The gated strategies were as follows: T cells (CD3^+^CD19^−^), B cells (CD3^−^CD19^+^), CD4 T cells (CD4^+^ CD8^−^), CD8 T cells (CD4^−^CD8^+^), and germinal center B cells (CD19^+^ FAS^+^CD38^−^).

### 2.10 Western blot analysis

Western blotting was utilized to analysis the expression level of E2A and PAX5 in spleen tissue. Briefly, to obtain total protein, the spleen tissues of mice were cut, ground and fully digested by utilizing RIPA lysis solution (Beyotime, P0013B) according to the manufacturer’s instruction. The BCA protein detection kit (Beyotime, P0012) was applied to determine the concentration of extracted protein in each sample. Equal amounts of protein were mixed with protein loading buffer (Beyotime, P0015L) and incubated at 100°C for 10 min, then separated by SDS-PAGE gel and transferred to a PVDF membrane. After blocking with 5% BSA in TBST for 1 h, the membrane was incubated with anti-E2A (Abclonal, 1:1,000), anti-PAX5 (Abclonal, 1:1,000) and GAPDH (Proteintech, 1:5000) primary antibodies overnight at 4°C. The membrane was washed with TBST for 3 times, and diluted secondary antibodies (Proteintech, 1:10000) were applied at room temperature for 1 h. After washing with TBST, the membranes were incubated with ECL solution (Servicebio) and detected using chemiluminescence. The relative expression of target protein was quantified by using ImageJ software (v1.8.0) and calculated using GAPDH as the internal reference.

### 2.11 Enzyme-linked immunosorbent assay

Serum antibody levels were determined using Mouse IgA, IgG and IgM ELISA Kit (Beyotime) according to the manufacturer’s instructions. Briefly, the required plates were placed in a 96-well frame. Add 100 μL of sample or different concentrations of standards into the wells and incubate for 120 min at room temperature. After washing the plate for 5 times, add horseradish peroxidase labelled mouse IgA antibody 100 μL/well and incubate for 20 min at room temperature away from light. After 5 washes, add TMB solution 100 μL/well and incubate for 15 min at room temperature away from light. Add the termination solution 50 μL/well, mix well and measure the A450 value immediately.

### 2.12 RNA sequencing

Total RNA was isolated from spleens by utilizing Trizol (CWBIO, China) reagents. MGISEQ-2000RS Kit for DNBseq 2000 was used to purify poly (A) transcripts and generate libraries. RNA-seq reads were aligned to the mouse genome (GRCm39) using HISAT2 with default parameters. The sequencing quality of the raw data was detected by utilizing FastQC (v0.10.1) software, and the raw data was filtered by Cutadapt (v1.9.1) to create clean data. The differential expressed genes (fold change ≥2, adjusted p ≤ 0.05) were obtained by utilizing DESeq2 analysis of three RNA-seq biological replicates from different donors. The volcano plot was generated by ggplot2 package in R. SXD regulated pathway analysis was performed by using gene ontology (GO) and Kyoto Encyclopedia of Genes and Genomes (KEGG) pathways in clusterProfiler. Gene Set Enrichment Analysis (GSEA) and Cell-type analysis of RNA-seq data were performed using WEB-based Gene Set Analysis Toolkit (https://www.webgestalt.org/, (Elizarraras et al., 2024)).

### 2.13 Network analysis

A total of 17 chemical metabolites of SXD were obtained based on LC-MS. The Simplified Molecular Input Line Entry System of each metabolite was imported into Swiss Target Prediction, Superpred, and STITCH databases for target prediction in turn, with the species set as “*Homo sapiens*”. The targets obtained from the three databases were combined to be the targets of SXD. The SXD targets were then intersected with the differential genes obtained by RNA-seq (P < 0.05, |fold change| >1.5), and 56 intersection targets were obtained. The Omicshare online platform (https://www.omicshare.com/) was utilized for the KEGG pathway enrichment analysis of intersection targets (*P* < 0.05). The 56 intersection targets were imported into the STRING database (http://string-db.org/) for protein-protein interaction (PPI) analysis, with species set to “*Homo sapiens*”, confidence set to 0.4, and the rest of the parameters were left at default settings. The PPI results were imported into Cytoscape 3.10.1, and 10 core targets were calculated using the “CytoHubba” plug-in. The Cytoscape3.9.1 software was utilized to map the KEGG pathway and core target interaction network, in addition to analyzing the topological properties of the network.

### 2.14 Reverse transcription real-time quantitative PCR (RT-qPCR)

Total RNA is extracted from splenic cells using TRIeasy™ Total RNA Extraction Reagent TRIeasy™ (YEASEN, 10606ES60) followed by chloroform phase separation. RNA is precipitated with isopropanol, washed with 75% ethanol, and dissolved in RNase-free water. Reverse transcription is performed using Hifair^®^ AdvanceFast 1st Strand cDNA Synthesis SuperMix for qPCR (YEASEN, 11156ES60). For qPCR, a 20 µL reaction mix is prepared with Hieff^®^ qPCR SYBR Green Master Mix (YEASEN, 11201ES08), target-specific primers, and cDNA template. Thermal cycling includes an initial denaturation, followed by 40 cycles of 95°C for 10 s and 60°C for 30 s. Data are normalized to 18S rRNA and analyzed using the ΔΔCt method for relative quantification. The primers used were as follows: Hif1-α forward, 5′-GTC​CCA​GCT​ACG​AAG​TTA​CAG​C-3′; Hif1-α reverse, 5′-CAG​TGC​AGG​ATA​CAC​AAG​GTT​T-3′; TLR4 forward, 5′- GAC​ACT​TTA​TCC​AGA​GCC​GTT​G -3′; TLR4 reverse, 5′- GGA​CTT​CTC​CAC​TTT​CTC​AAG​G -3′; MMP-9 forward, 5′-GTA​CCA​CGG​CCA​ACT​ACG​AC-3′; MMP-9 reverse, 5′-GCC​TTG​GAA​GAT​GAA​TGG​AA-3′; Nr3C1 forward, 5′- GCA​GTG​GAA​GGA​CAG​CAC​AA-3′; Nr3C1 reverse, 5′- GAG​ACT​CCT​GCA​GTG​GCT​TG-3′; Kdr forward, 5′-ATC​CCT​GTG​GAT​CTG​AAA​CG-3′; Kdr reverse, 5′-CCA​AGA​ACT​CCA​TGC​CCT​TA-3′; 18 s forward, 5′-ACT​TTG​GCA​TTG​TGG​AAG​GG-3′; 18 s reverse, 5′-CGG​ACA​CAT​TGG​GGG​TAG​GA-3′.

### 2.15 Statistical analysis

All data were presented as mean ± SD. The statistically significant differences between virous groups were analyzed by one-way ANOVA by GraphPad Prism version 8. Differences between groups at p < 0.05 were considered statistically significant.

## 3 Results

### 3.1 Chemical analysis of SXD extracts

**FIGURE 1:**
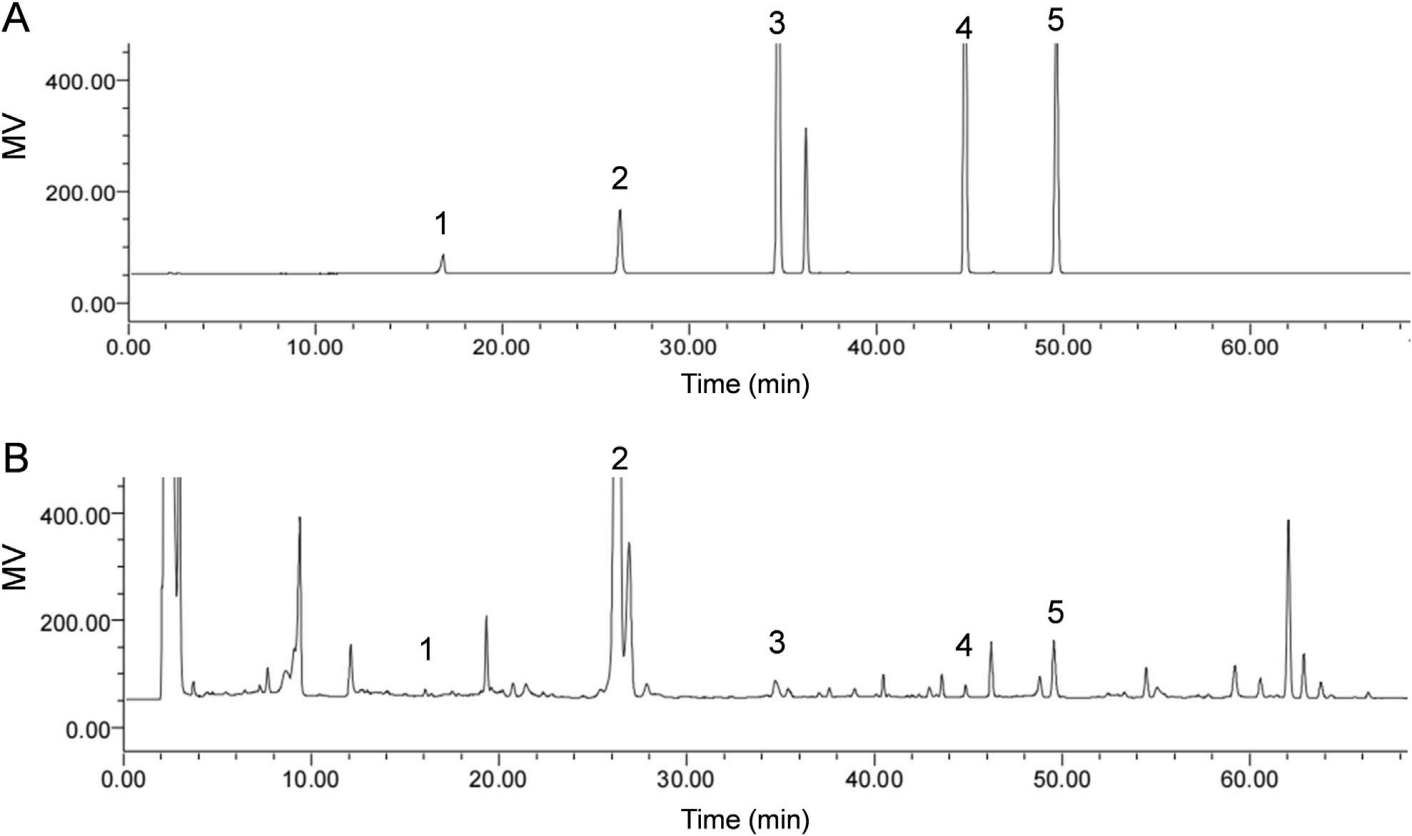
Identification of chemical metabolites of SXD. HPLC chromatograms analysis of the **(A)** standard solutions and **(B)** SXD extracts. 1, Iso ferulicacid; 2, Timosaponin BII; 3, Platycodin D; 4, Astragaloside; 5, Saikosaponin A.

In order to identify the metabolites of SXD, we performed the UHPLC/MS/TOF to analysis the aqueous extraction from SXD (Supplementary Figure S1). A total of seventeen metabolites were identified in SXD under the negative mode, comprising nine terpenoids, five flavonoids and three phenylpropanoids (Supplementary Table S1). Among these, the signature metabolites of SXD had been identified according to the standard substances, including astragaloside, iso ferulicacid, saikosaponin A, platycodin D and timosaponin BII (Figure 1), which was consistent with previous reports (Zhang et al., 2014; Huang et al., 2021).

### 3.2 Amelioration of immunosuppression in the spleen and thymus by SXD administration

**FIGURE 2:**
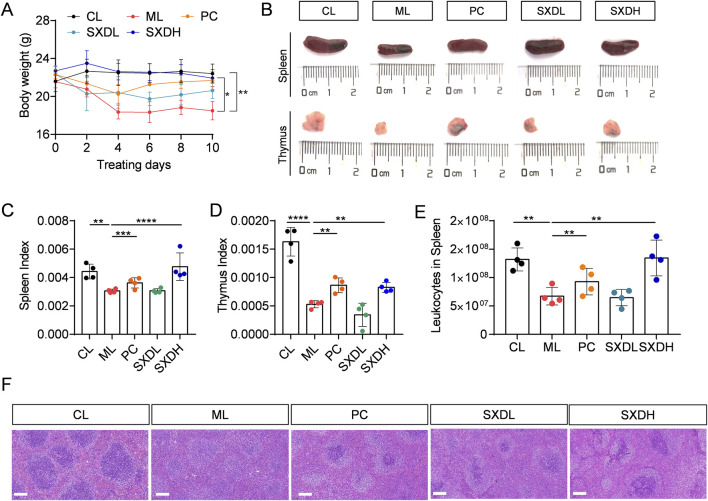
Protection effect of SXD on CTX-induced mice. **(A)** The body weight of CTX-treated mice during SXD administration; **(B)** The spleen and thymus photograph in each group; **(C)** Spleen index; **(D)** Thymus index. **(E)** The cell number of leukocytes in spleen. **(F)** Hematoxylin-eosin staining of spleen sections. Scale bar = 100 μm. CL: administrated with saline; ML: intraperitoneally administrated with cyclophosphamide; PC: intraperitoneally administrated with cyclophosphamide and followed by levamisole hydrochloride at 20 mg/kg; SXD-L, M and H intraperitoneally administrated with cyclophosphamide and followed by SXD treatment, respectively; N = 4 for each group, **: P < 0.01; ***: P < 0.001; ****: P < 0.0001.

To investigate the immune modulation of SXD, a CTX-induced immunosuppression mouse model was established. Compared to CL mice, CTX-treated mice exhibited weight loss (Figure 2A), smaller spleen and thymus (Figure 2B), and disruption of spleen structure (Figure 2F). However, a significant weight recovery was observed in mice from the PC and SXDH groups in comparison to those from the ML group (Figure 2A). SXDH treatment significantly increased spleen and thymus index in CTX-treated mice (Figures 2B–D). Furthermore, SXDH treatment led to a substantial increase in the number of leukocytes in the spleen, concomitant with the restoration of the splenic follicular structure (Figures 2E,F). However, lower doses of SXD did not significantly improve organ indices, and these data do not support a clear dose-dependent relationship. These results indicate that high-dose SXD treatment significantly ameliorated CTX-induced spleen and thymus damage in mice.

### 3.3 SXD improved CTX-induced reduction of B cell population in spleen

**FIGURE 3:**
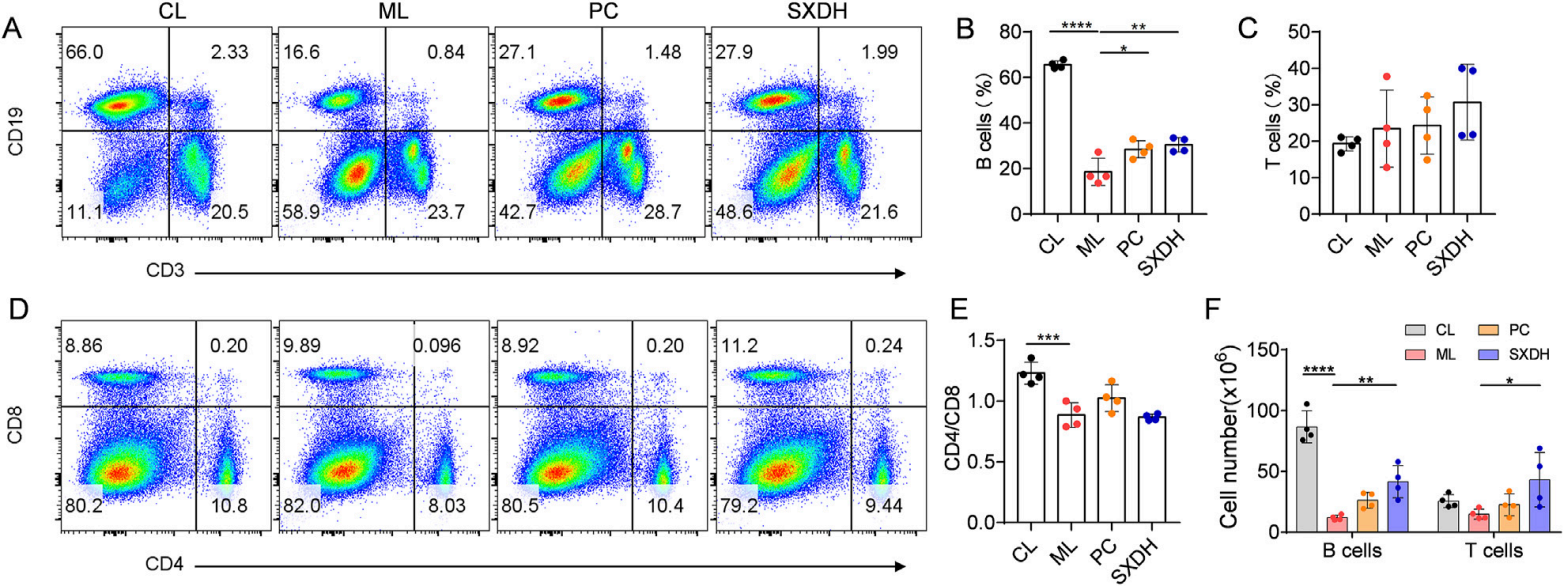
The immune-boosting effect of SXD on lymphocyte subsets in CTX-induced mice. **(A)** Representative flow cytometric images showing T cells and B cells in indicated mice. **(B,C)** Quantitative analysis for relative percentage of B cells **(B)** and T cells **(C)** in each group. **(D)** Representative flow cytometric images showing CD4 and CD8 T cells in indicated mice. **(E)** The CD4/CD8 cell ratio in spleen. **(F)** Quantitative analysis for cell number of B cells and T cells in spleen. Data are representative of plots from two independent experiments. N = 4 for each group, *: P < 0.05, **: P < 0.01; ***: P < 0.001; ****: P < 0.0001.

The predominant immune cells within the spleen are B cells and T cells. To investigate the protection effect of SXD on splenic cells, we conducted a flow cytometry analysis of the population of T cells and B cells in spleen. In comparison to the control group, the percentages of CD19^+^ B cells were found to be significantly reduced in the ML group (Figures 3A,B). Treatment with SXD resulted in an increase in the proportion of B cells (Figure 3B). It is noteworthy that the proportion of T cells remained consistent across all groups of mice (Figure 3C). Further exploration of the internal subpopulations of T cells revealed that CTX treatment led to a decrease in the ratio of CD4/CD8 (Figure 3D). SXD treatment was unable to restore the imbalance in the CD4/CD8 ratio (Figure 3E), suggesting that SXD was unable to regulate the imbalance of the internal subpopulations of T cells. The analysis of T and B cell number in the spleen demonstrated that SXD treatment resulted in a significant increase in these cell types (Figure 3F). These results indicated that SXD ameliorates the CTX-induced decrease in T and B cell numbers and significantly increases the proportion of B cells.

### 3.4 SXD promotes B-cell mediated humoral immunity

**FIGURE 4:**
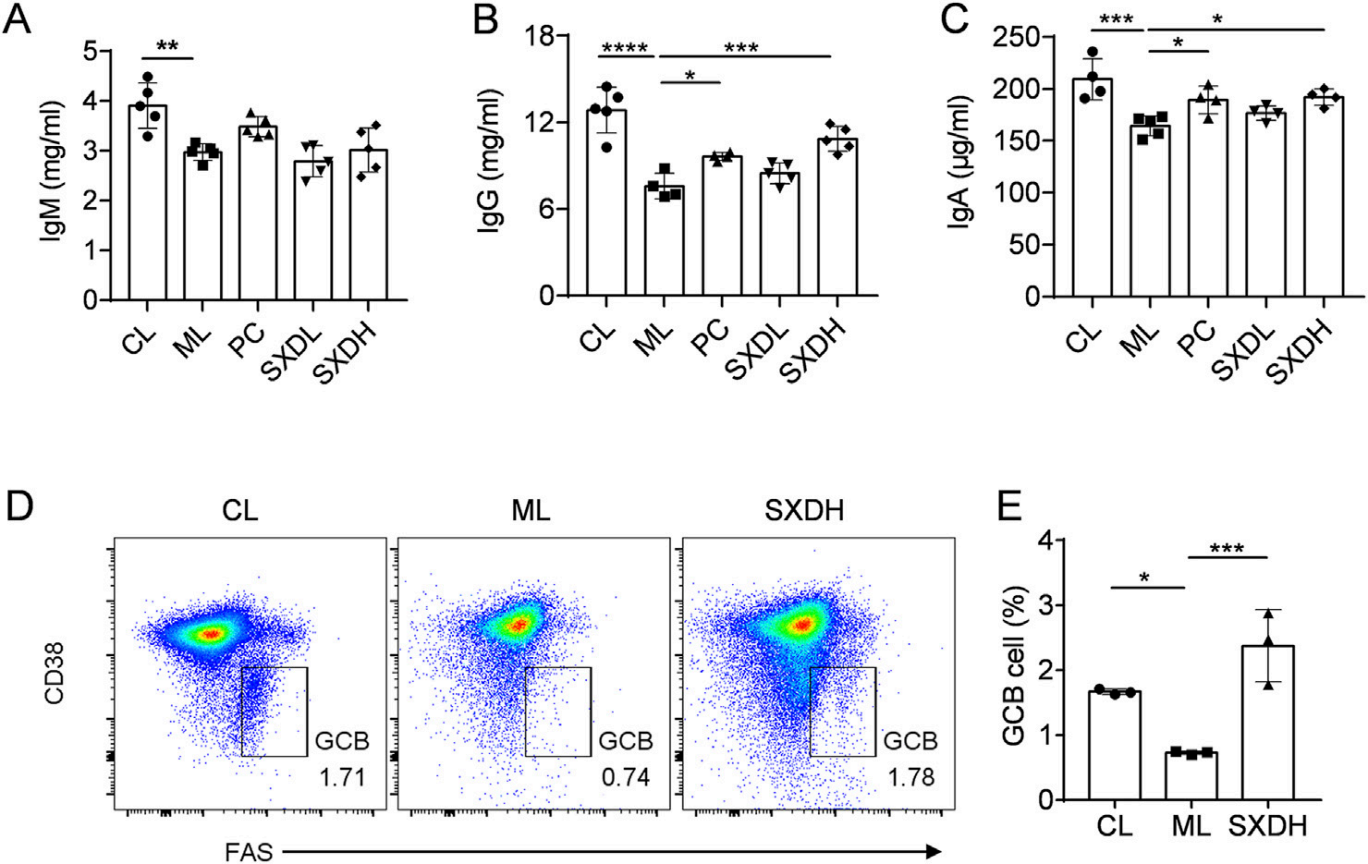
SXD treatment promotes the humoral immune response in CTX-induced mice. **(A–C)** The quantitative test of IgM, IgG and IgA in serum. **(D)** Representative flow cytometric images showing germinal center B cells in model and SXD-treated group. **(E)** Quantitative analysis for relative percentage of germinal center B cells (CD19^+^CD38^−^FAS^+^). Data are representative of plots from two independent experiments. N = 3–5 for each group, *: P < 0.05, **: P < 0.01; ***: P < 0.001; ****: P < 0.0001.

B lymphocytes are the source of protective antibodies (also known as immunoglobulins), which mainly mediate humoral immunity (Wang et al., 2020). To gain further insight into the impact of SXD on humoral immunity in immunosuppressed mice, we undertook the ELISA analysis to detect the antibody levels. SXD administration also led to a significant elevation in serum IgA and IgG levels in comparison to the model group, while no notable impact was observed on IgM levels (Figures 4A–C). Germinal center (GC) B cells are essential for the production of high-affinity antibodies, so we further examined the proportion of GCB cells in mesenteric lymph nodes. The results of the flow cytometry analysis demonstrated a statistically significant decrease in the proportion of GCB cells in the ML group compared to the CL group. However, the administration of SXD resulted in a significant recovery of GCB cells (Figures 4D,E). These results shows that SXD enhances B cell activation and antibody response in immunosuppression mice.

### 3.5 SXD regulates the B cell receptor and immunoglobulin-related pathways

**FIGURE 5:**
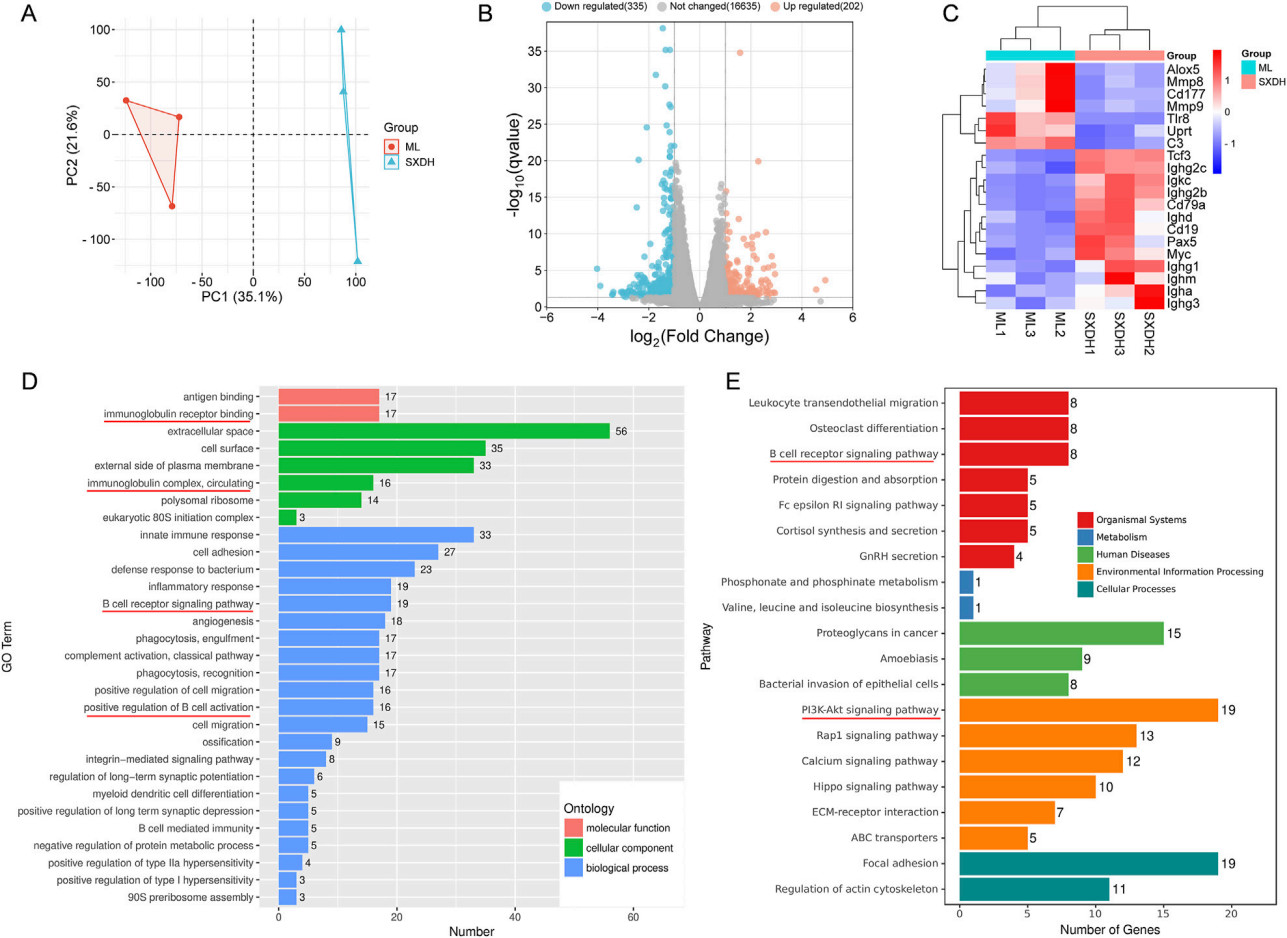
RNA-seq analysis of differential gene expression in spleen between SXDH and ML group. **(A)** PCA of variation between SXDH and ML group. **(B)** Volcano plots of SXDH vs. ML group. FDR ≤ 0.05, |Fold change| ≥ 2. **(C)** Heatmap shows differentially expressed genes. **(D)** GO enrichment analysis of differentially expressed genes. **(E)** KEGG enrichment analysis of differentially expressed genes.

In order to ascertain the mechanism by which SXD exerts an immune-boosting effect in the context of CTX-induced immunosuppression, we conducted the RNA sequencing analysis of ML and SXDH splenic cells. The results of the principal component analysis indicated a significant divergence in the gene expression between the ML and SXDH treatment groups (Figure 5A). The RNA-seq results demonstrated that 202 genes were upregulated and 335 genes were downregulated in spleen cells in response to SXD treatment under immunosuppression conditions (Figure 5B). As showed by the gene expression heatmap, SXD treatment resulted in the upregulation of B-cell marker and immunoglobulins, including Cd19, Cd79a and Ighg1. Concurrently, SXD treatment led to the downregulation of inflammation-related genes, such as Cd177 and Alox5 (Figure 5C). The Gene Ontology enrichment analysis of the differential genes revealed that the pathways regulated by SXD included immunoglobulin receptor binding, B cell receptor signaling pathway and positive regulation of B cell activation (Figure 5D). The Kyoto Encyclopedia of Genes and Genomes enrichment analysis of the differential genes revealed that SXD-regulated genes were implicated in the B-cell receptor signaling pathway and the PI3K-AKT signaling pathway (Figure 5E). These findings indicate that SXD treatment alters the B cell receptor signaling pathway.

### 3.6 SXD increased the proliferation of splenic lymphocytes in immunosuppression mice

**FIGURE 6:**
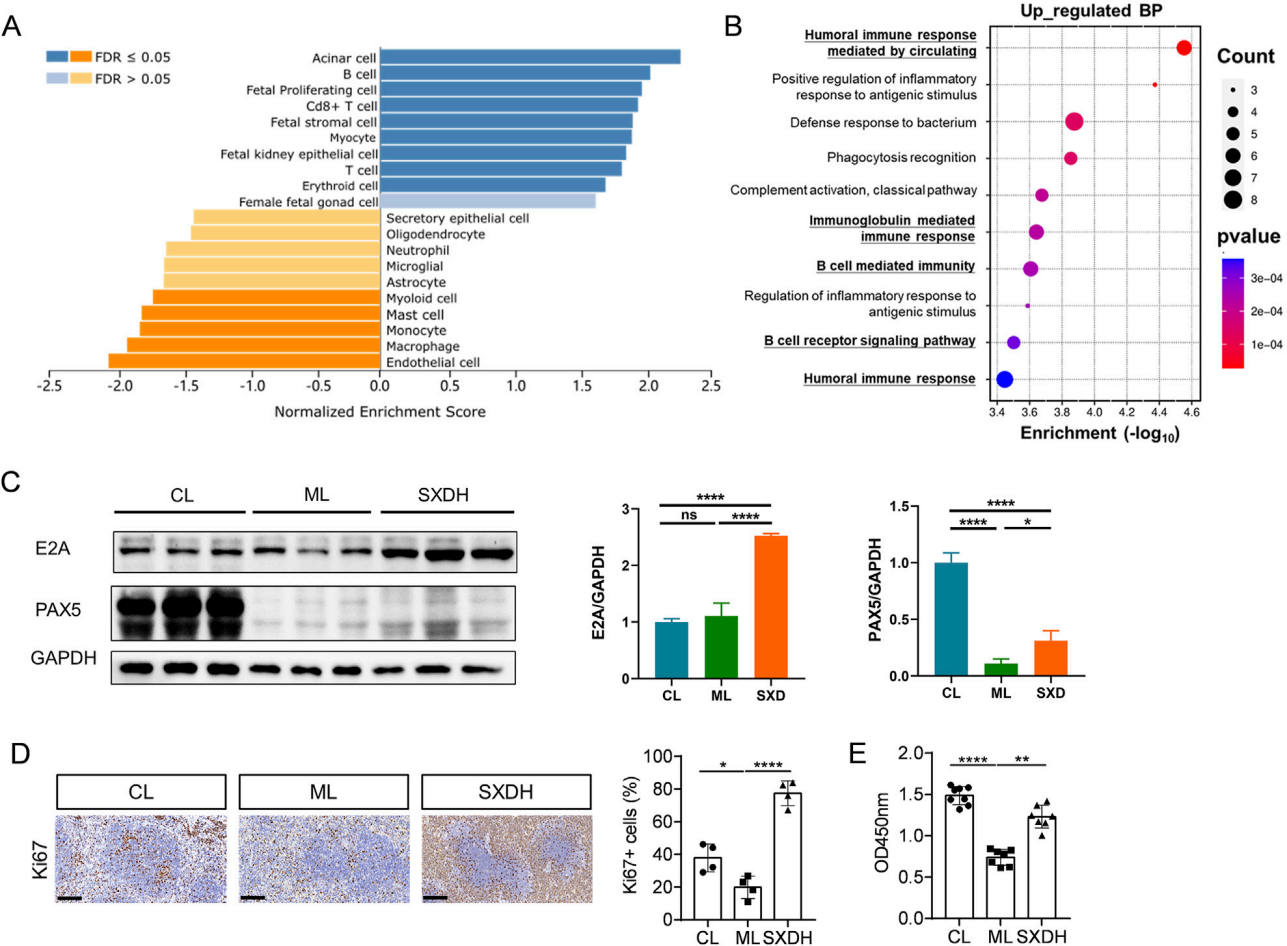
SXD treatment promoted the expression of B cell proliferation and differentiation factors. **(A)** Cell-type analysis of RNA-seq data using WEB-based Gene Set Analysis Toolkit. **(B)** GO enrichment analysis of SXD upregulated genes. **(C)** The Western blotting results of E2A and PAX5. **(D)** Immunohistochemistry images of Ki67 protein expression in spleen tissues. Scale bar = 100 μm. **(E)** Analysis of LPS-induced splenic lymphocytes proliferation *in vitro* using CCK8. *: P < 0.05, **: P < 0.01, ***: P < 0.001. ****: P < 0.0001.

To further analyze the regulatory effects of SXD on splenic immune cells, we processed the data from RNA sequencing using the WEB-based Gene Set Analysis Toolkit. The cell-type analysis showed that SXD treatment elevated the proportion of T and B cells, and decreased the proportion of myeloid cells (Figure 6A). The Gene Ontology enrichment analysis of upregulated genes revealed that SXD significantly upregulated B-cell and humoral immunity-related pathways (Figure 6B). The RNA-seq results demonstrated that SXD administration led to a notable elevation in the expression levels of B-cell differentiation-related genes, including Pax5 and Tcf3 (Figure 5C). The results of protein immunoblotting provided further confirmation that SXD treatment elevated protein expression of E2A (encoded by Tcf3) and PAX5 transcription factors (Figure 6C). The transcription factors Pax5 and Tcf3 are critical for controlling B cell differentiation (Calderón et al., 2021; Boast et al., 2023). The elevated expression of Pax5 and Tcf3 indicates that SXD treatment facilitates B cell differentiation. Furthermore, SXD treatment also increased the expression of the transcription factor Myc (Figure 5C), which has been demonstrated to promote B cell proliferation (Finkin et al., 2019). To further verify whether SXD has the function of promoting lymphocyte proliferation, we examined the expression of Ki67 in the spleen. In comparison to the CL group, the percentage of Ki67^+^ cells in the spleen reduced in the ML group, whereas it was increased in the SXD administration group (Figure 6D). To further verify the capacity of SXD to promote lymphocyte proliferation, splenic lymphocytes were isolated from each group and stimulated by LPS. The results demonstrated that LPS-induced splenic lymphocyte proliferation was significantly reduced in the ML group, but enhanced after SXD administration (Figure 6E). These results demonstrated that SXD treatment could promote splenic lymphocytes proliferation under immunosuppression condition.

### 3.7 SXD inhibits hypoxia and aging pathway

**FIGURE 7:**
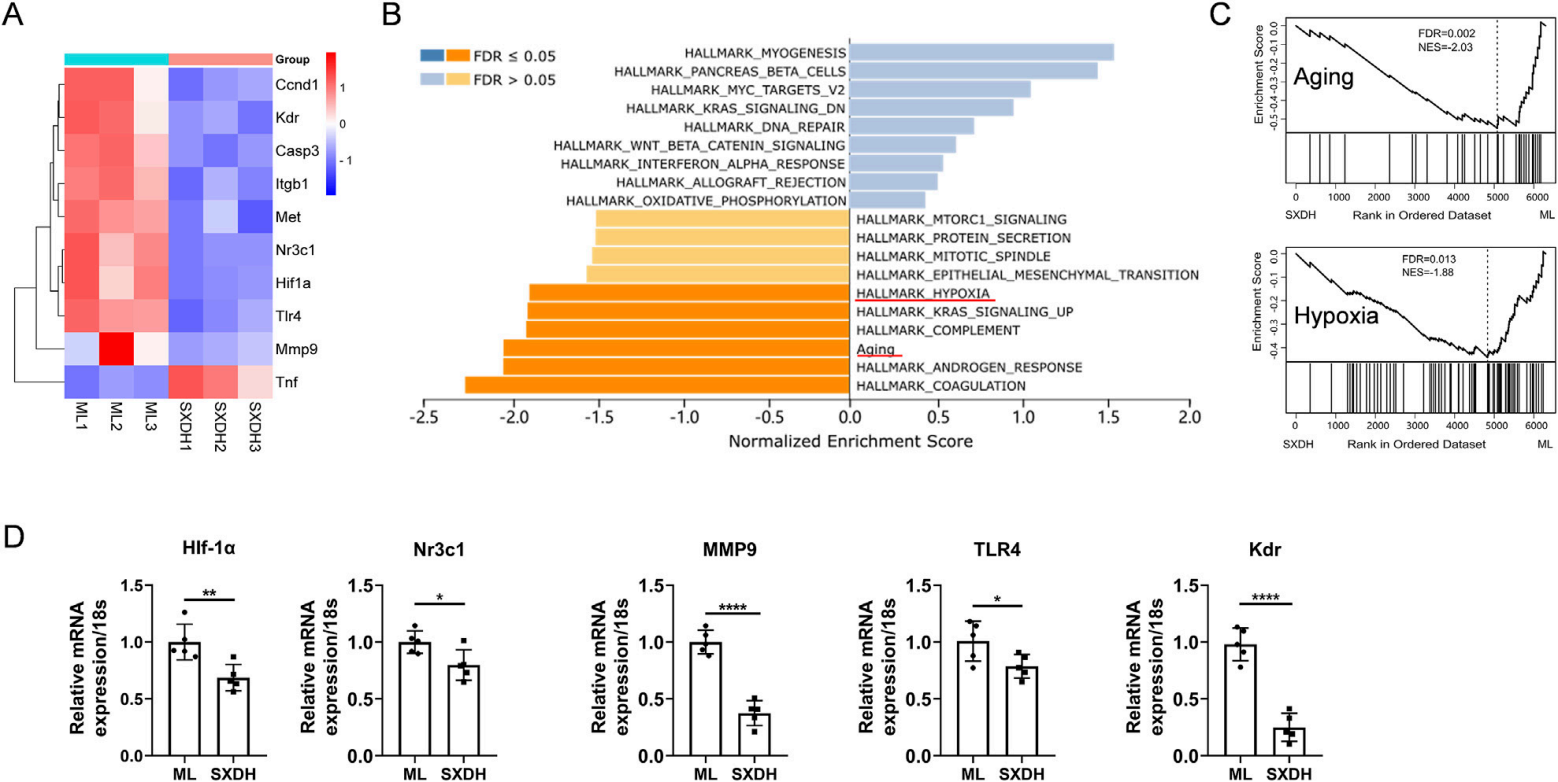
SXD treatment downregulates the expression of hypoxia and aging gene sets. **(A)** Differential expression of the indicated genes in RNA sequencing data. **(B)** Gene Set Enrichment Analysis of RNA-seq data using WEB-based Gene Set Analysis Toolkit. **(C)** Enrichment analysis of Aging and hypoxia gene sets based on RNA sequencing results. **(D)** RT-qPCR analysis of the expression of indicated genes. *: P < 0.05, **: P < 0.01, ***: P < 0.001. ****: P < 0.0001.

To further reveal the complex mechanism of action of SXD, we performed network analysis combined with transcriptomics analysis. The predicted SXD targets were taken as intersections with the differential genes from RNA sequencing, and 56 intersecting targets were obtained (Supplementary Figure S2A). The 56 intersecting targets were further analyzed for protein-protein interactions and 10 core targets were obtained, including HIF1A, MMP9 and CASP3 (Supplemetary Figures S2B,C). The signaling pathways regulated by these core genes include PI3K-Akt, MAPK, HIF-1, and TNF signaling pathways (Supplemetary Figures S2D). The results of RNA sequencing showed that the expression of nine core genes was downregulated under SXD treatment, except TNF (Figure 7A). This finding suggests that SXD treatment primarily downregulates gene expression, a conclusion that is consistent with the results presented in the volcano plot (Figure 5B). We further analyzed the regulation of SXD on signaling pathways based on RNA sequencing data using GSEA enrichment (Figure 7B). We added a previously reported Aging gene set to the HALLMARK gene set. The analysis of Aging gene set has been demonstrated to be a reliable method for identifying the senescence status of tissues and cells (Saul et al., 2022). Interestingly, SXD treatment downregulated both hypoxia and senescence pathways in our RNA sequencing results (Figure 7C). To corroborate the findings from RNA sequencing, we performed RT-qPCR analysis to assess the expression levels of key genes within the hypoxia- and senescence-related gene sets. Our results demonstrated that treatment with SXD markedly suppressed the expression of these genes, further supporting the RNA sequencing data (Figure 7D). These results suggest that SXD extract inhibits hypoxia and senescence core genes, which in turn improves the immunosuppression of spleen.

## 4 Discussion

In TCM theory, SXD is a commonly used clinical treatment for a syndrome known as “atmospheric depression,” which presents with a constellation of symptoms including chest tightness, shortness of breath, palpitations, dizziness, and forgetfulness. It is noteworthy that individuals infected with the SARS-CoV-2 virus also experience fatigue, shortness of breath, and cognitive impairment within 3 months of infection (Subramanian et al., 2022; Whitaker et al., 2022). It has been demonstrated that an excessive anti-inflammatory response and low immune function are the mechanisms that underpin the sequelae of SARS-CoV-2 infection (Merad et al., 2022). Additionally, individuals diagnosed with lung cancer frequently present with profound fatigue, dyspnea, loss of appetite, and immunosuppression following chemotherapy (Luo et al., 2024). Individuals with impaired immune systems, whether as a result of chemotherapy or infection with the SARS-CoV-2 virus, present with symptoms that are consistent with atmospheric depression syndrome. The aforementioned studies indicate that SXD may enhance immune function in immunocompromised patients.

CTX is widely used to create immunosuppression mouse model (Bushmeleva et al., 2024; Zhao et al., 2024). CTX inhibits the proliferation, survival and differentiation of immune cells. Therefore, CTX is also used to treat autoimmune diseases, such as systemic lupus erythematosus. The findings of both previous studies and our own research indicate that CTX exerts a more pronounced inhibitory effect on B cells than on T cells (Li Y. et al., 2023). B cells represent the primary lymphocytes involved in humoral immunity. Consequently, the suppression of B cells will have a significantly detrimental impact on humoral immunity (Tokunaga et al., 2019). Our results show that SXD significantly increases spleen index and increase B cell population. The results of RNA sequencing elucidate that SXD significantly activates the BCR pathway and upregulates the transcription factors Pax5, Tcf3 and Myc. These results indicate that SXD provokes BCR signal and prompts B cell-mediated humoral immunity.

The development and proliferation of B cells are controlled by precise transcriptional regulation. B cells develop from hematopoietic stem cells in the bone marrow and are activated to differentiate into plasma cells that secrete antibodies against infection when stimulated by antigens (Eibel et al., 2014). The transcription factor Tcf3 is essential for the differentiation of committed B-lineage cells. B-cell development in Tcf3 deficient mice was blocked in the Pre-pro B cell stage (Kee et al., 2000). The germinal center (GC) is the place where B cells produce high-affinity antibodies, and GC-B cell development is abnormal in Tcf3 deficient mice (Wöhner et al., 2016). In B cells, Tcf3 further upregulates the expression of transcription factor Pax5 (Kwon et al., 2008). PAX5 promotes the expression of B cell characteristic genes and inhibits the expression of non-B cell genes, which is essential for the development and maintenance of B cells (Calderón et al., 2021). Mature B cells dedifferentiate into uncommitted progenitors in Pax5-deleted mice (Medvedovic et al., 2011). In addition, Pax5 also promotes B-cell antibody diversity by regulating V-DJ rearrangement (Ebert et al., 2015). We found that the expression of Tcf3 and Pax5 was significantly increased in SXD-treated mouse spleen cells, hinting that SXD promotes B cell differentiation by up-regulating the expression of Tcf3 and PAX5. Our results also revealed that SXD upregulates the expression of the transcription factor Myc, which is induced by signals such as BCR signals. Myc is essential for the proliferation of activated B cells and the formation of GC B cells (Luo et al., 2018; Finkin et al., 2019). In our results, SXD significantly upregulates the expression of Myc, which is consistent with the result of *in vitro* experiment that SXD treatment accelerates the proliferation of mouse spleen cells. Therefore, we speculate that SXD promotes B cell differentiation, maintenance and proliferation by up-regulating the expression of Tcf3, Pax5 and Myc.

B cell homeostasis depends on a diverse microenvironment *in vivo*. Both excessive activation of hypoxia and senescence signals promote apoptosis and abnormal differentiation of B cells (Cho et al., 2016; Burrows et al., 2020; Li X. et al., 2023). Despite the fact that RNA sequencing data demonstrated that SXD treatment significantly increased the expression of B-cell differentiation factors Pax5, Tcf3 and BCR signaling pathways, RNA sequencing complemented by network analysis also demonstrated that SXD metabolites specifically target hypoxia- and cellular senescence-related signaling pathways. Consequently, it is proposed that SXD extract may directly inhibit the hypoxia and cellular senescence signaling pathways to improve the microenvironment in the spleen and indirectly promotes B cell homeostasis and enhances humoral immunity.

Our study highlights the regulatory effects of SXD on B cells, but it is crucial to consider the broader immune landscape that may contribute to its therapeutic efficacy. Immune suppression and modulation are orchestrated by a complex interplay of various immune cell types, including T cell subsets (e.g., CD4^+^ T helper cells, CD8^+^ cytotoxic T cells, and regulatory T cells) and macrophages (e.g., M1/M2 polarization). Future research should aim to explore the impact of SXD on these populations to better understand its immunomodulatory mechanisms. Additionally, high dimensional immune profiling techniques, such as CyTOF and single-cell RNA sequencing, could be employed to assess the global effects of SXD on immune cell populations. Additionally, investigating the molecular pathways through which SXD regulates these cells would provide deeper insights into its multi-target mechanisms.

Our study was conducted based on previous clinical studies (Jiang et al., 2022; Jiang and Zhao, 2024). Following 6 weeks of combination therapy with SXD and cisplatin, cancer-related fatigue showed significant improvement. Notably, the SXD-cisplatin combination demonstrated lower incidence of toxic side effects compared to cisplatin monotherapy, along with elevated T lymphocyte proportions (Jiang et al., 2022). These findings suggest synergistic therapeutic effects between SXD and standard chemotherapy regimens. While SXD exhibited efficacy in both human and murine chemotherapy models, its clinical application emphasizes TCM syndrome differentiation. Current clinical trials exclusively enrolled patients with Qi deficiency syndrome, whereas murine models inherently lack capacity for TCM constitutional classification. The therapeutic advantages of SXD might be more pronounced in murine models simulating Qi deficiency with concomitant immunosuppression. However, clinical efficacy in chemotherapy populations without overt Qi deficiency requires further investigation. Comprehensive evaluation of SXD’s long-term safety profile remains imperative. Two existing clinical trials in chemotherapy patients with treatment durations of 6 weeks (n = 102) and 2 months (n = 80) respectively reported no significant toxicity (Jiang et al., 2022; Jiang and Zhao, 2024). Furthermore, combination therapy with SXD significantly reduced incidences of chemotherapy-associated hepatotoxicity, gastrointestinal reactions, and myelosuppression compared to chemotherapy alone. These observations indicate that SXD administration for 1–2 months demonstrates acceptable safety, yet extended usage beyond this period warrants systematic pharmacovigilance. Collectively, these results position SXD as a therapeutic agent with promising clinical translation potential.

In summary, our results show that SXD significantly enhances the B-cell-mediated humoral immune response, including increasing the proportion of lymphocytes in the spleen and upregulating the expression of antibody genes. RNA sequencing reveals the partial mechanism that SXD regulates B cells through the activation of BCR pathway and the inhibition of hypoxia and cellular senescence signaling pathways. The findings of our study provide a theoretical basis for the broader clinical application of SXD.

## Data Availability

The raw data of RNA-seq reported in this paper have been deposited in the Genome Sequence Archive (Chen et al., 2021) in National Genomics Data Center, China National Center for Bioinformation / Beijing Institute of Genomics, Chinese Academy of Sciences (GSA: CRA017537) that are publicly accessible at https://ngdc.cncb.ac.cn/gsa/browse/CRA017537.
