# Supplementary material for: Shengxian decoction alleviates cyclophosphamide-induced immunosuppression via improving B cell-mediated immune responses
Source: Front Pharmacol. 2025 Apr 23;16:1565451. doi: 10.3389/fphar.2025.1565451 (PMC12055854; doi:10.3389/fphar.2025.1565451)
Supplement: Supplementary file 1 [file DataSheet1.docx]

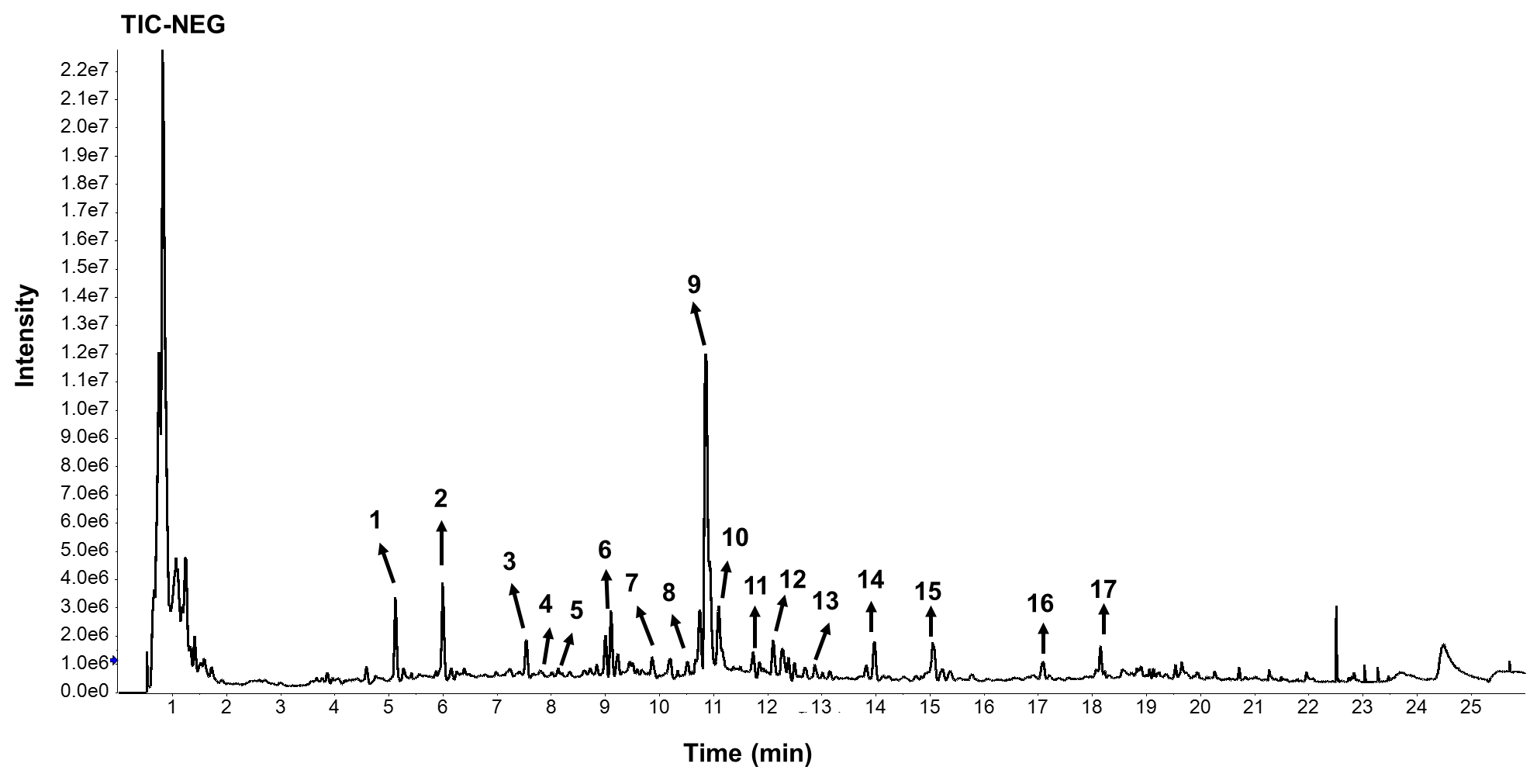
Supplementary figures

Figure. S1 Chromatograms of UHPLC/MS/TOF of SXD extraction. The compounds are listed in Table S1.


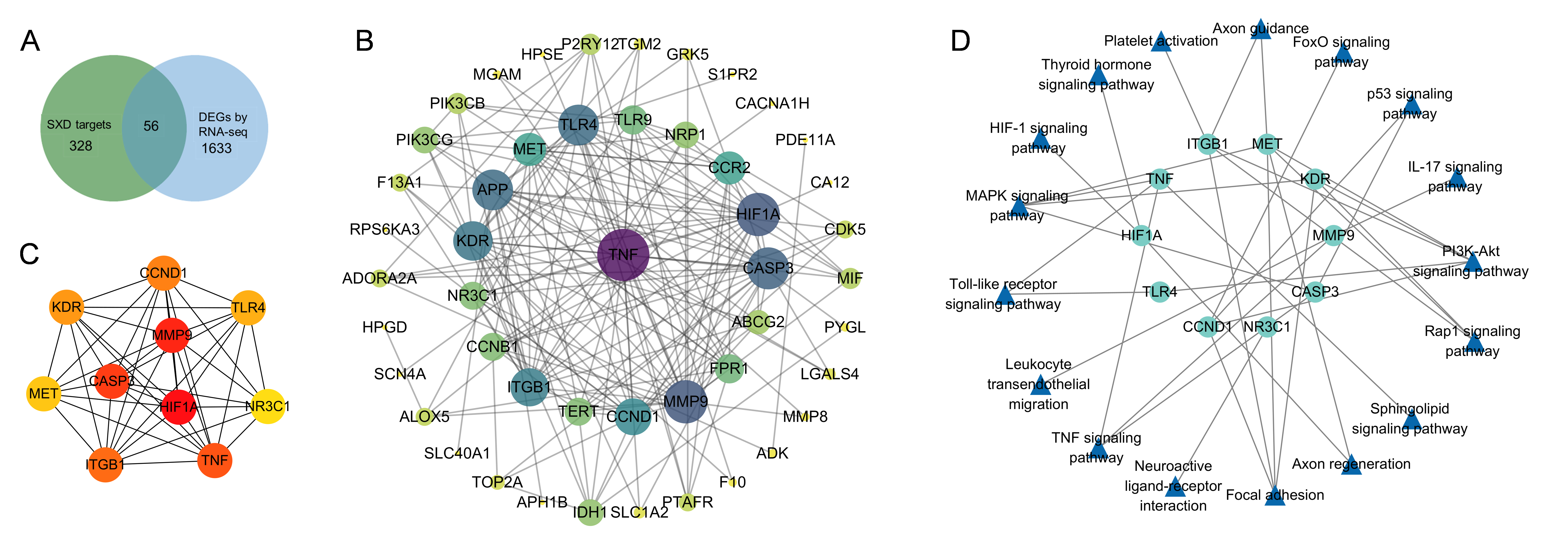


Figure. S2 Network analysis of SXD target pathways. (A) Venn diagram showing the intersection of SXD target genes and differentially expressed genes (DEGs, P<0.05, |fold change| >1.5) obtained by RNA sequencing. (B) Pro-tein-Protein Interaction Network of 56 intersecting targets. (C) 10 core targets obtained from 56 intersecting targets. (D) Network of 10 core targets interacting with the KEGG pathway.

Table S1. The main compound of aqueous extraction from SXD

| NO. | RT (min) | Neutral mass (m/z) | Formula | Identification | Measured mass (m/z) | Error (ppm) | Source |
| --- | --- | --- | --- | --- | --- | --- | --- |
| 1 | 5.12 | 583.13019 | C25H27O16 | Neomangiferin | 583.12936 | 1.4 | ZhiMu |
| 2 | 5.99 | 421.07743 | C19H17O11 | Mangiferin | 421.07654 | 2.1 | ZhiMu |
| 3 | 7.53 | 445.11586 | C22H21O10 | Calycosin 7-O-glucoside | 445.11292 | 6.6 | HuangQi |
| 4 | 7.79 | 193.05037 | C10H9O4 | Ferulicacid | 193.04954 | 4.3 | ShengMa |
| 5 | 8.13 | 193.05027 | C10H9O4 | Iso Ferulicacid | 193.04954 | 3.8 | ShengMa |
| 6 | 9.0 | 283.06092 | C16H11O5 | Calycosin | 283.06010 | 2.9 | HuangQi |
| 7 | 9.86 | 935.48697 | C45H75O20 | Timosaponin E | 935.48462 | 2.5 | ZhiMu |
| 8 | 10.74 | 431.09891 | C21H19O10 | Cimicifugic acid F | 431.09727 | 3.8 | ShengMa |
| 9 | 10.85 | 919.48859 | C45H75O19 | Timosaponin B II | 919.48971 | -1.2 | ZhiMu |
| 10 | 11.09 | 919.49192 | C45H75O19 | Timosaponin A1 | 919.48971 | 2.4 | ZhiMu |
| 11 | 11.73 | 267.06645 | C16H11O4 | Formononetin | 267.06519 | 4.7 | HuangQi |
| 12 | 12.10 | 1223.57031 | C57H91O28 | Platycodin D | 1223.56914 | 1.0 | JieGeng |
| 13 | 12.87 | 1265.57845 | C59H93O29 | Platycodin A | 1265.57970 | -1.0 | JieGeng |
| 14 | 13.97 | 901.47964 | C45H73O18 | Anemarsaponin B | 901.47914 | 0.6 | ZhiMu |
| 15 | 15.02 | 783.45748 | C41H67O14 | Astragaloside | 783.45253 | 6.3 | HuangQi |
| 16 | 17.08 | 757.43775 | C39H65O14 | Timosaponin I | 757.43688 | 1.1 | ZhiMu |
| 17 | 18.15 | 779.45687 | C42H67O13 | Saikosaponin A | 779.45762 | -1.0 | ChaiHu |
